# Book Reviews

**Published:** 1799-03

**Authors:** 


### MEDICINE AND SURGERY.

*A Collection of Teſtimonies reſpecting the Treatment of the Venereal Diſeaſe by Nitrous Acid;* publiſhed by Thomas Beddoes, *M. D.* 8vo. XVII. and 277 pp. *London.* Johnſon. 1799.

This collection is the reſult of Dr. Beddoes's indefatigable exertions in the cauſe of ſcientific truth and medical improvement, particularly as it ſtands connected with the important ſubject of the nitrous acid, as a remedy in lues. It exhibits in one view the collective maſs of evidence for, and againſt, the uſe of this powerful agent, in that diſorder. The unqueſtionable facts here adduced, afford an extenſive and very intereſting view of the nitrous acid, and analogous antiſyphilitics, as hitherto obſerved and detailed by a conſiderable number of medical gentlemen, no leſs eminent for their abilities and experience, than reſpectable for the ſtations they occupy in ſociety. Among theſe, it will be ſufficient to mention the reſpective names of Drs. Trotter, Rollo, Geach, Swediauer, Currie, Carmichael, Carrick, &c.

The teſtimonies thus collected and exhibited appear, on the whole, to be conſiderably in favour of the new medicine; although impartiality obliges us to obſerve, that there exiſts ſome diſagreement in the evidence, reſpecting the ſenſible changes in the patients, on whom theſe remedies were tried with ſucceſs. Such diſcordance, however, is not a novel phenomenon in the practice of medicine; and, conſidering the precipitate manner in which new remedies are *generally* adminiſtered; the want of proper attention to the peculiar habits, conſtitutions, and prediſpoſing cauſes, and other concomitant circumſtances of the patient—imperfections not ſeldom obſervable among a certain claſs of practitioners, (ſuch, eſpecially, as have too many inveterate caſes under their care *at one time*); we have reaſon, conſidering theſe circumſtances, to be highly ſatisfied with the reports of the *inefficacy of the acid*, as indicated by certain ſuperficial writers.

Indeed, the account given by Dr. Currie is ſo ſatisfactory and concluſive, that a few extracts from the maſterly and judicious letter * of that excellent phyſician will, we ſhould imagine, be deemed ſufficient to confirm the favourable

* See page 129—136 of Dr. Beddoes's '*Collection*.'

favourable opinion almoſt generally entertained on this valuable acceſſion to the ſtock of the *Materia Medica*.—We have no intention, however, to take any particular notice here of the ſingular controverſy now ſubſiſting on that ſubject.—

"The encouragement ariſing from the three caſes ‡ juſt mentioned, led me to try the nitric acid in a variety of other caſes. In ſome of theſe, my ſucceſs has apparently been complete; in others, there has been evident benefit, without a perfect cure; and in others, it has ſeemed to fail entirely. It is not a little curious, that in ſome of the caſes in which I have ſucceeded, the ſymptoms were what are called *ſecondary*, and the diſeaſe *in its moſt rooted and obſtinate ſtate*."

The uncommon ſucceſs with which the uſe of this acid was attended in another patient of Dr. Currie's, named *Ekins*, claims further and particular notice.—"We preſcribed (ſays the Doctor) the nitrous acid, and his ſufferings abated from the third day; and being continued, the thickening of the pericranium and the node of the tibia entirely diſappeared, with all his other ſymptoms. He took the nitrous acid, in all, to the quantity of eight ounces, in eight gallons of water, which he drank in ſixty days.

"Elkins has been nearly a year diſcharged, and has never had any return of his complaints. This caſe has made ſome noiſe, and I have endeavoured to attract the attention of ſeveral of my brethren to it, as deciſive of the influence of the acid in this deſtructive diſeaſe.

"In the quantities in which I have preſcribed the nitric acid, it has been *uniformly* ſalutary to the conſtitution; in this reſpect, its action contraſting very happily with that of mercury.

"I have experienced the effects of the nitric acid in complaints of the ſtomach, hypochondriaſis, aſthma, and ſome other diſeaſes, as well as in the hepatitis."

The ſecond diviſion of Dr. Beddoes's Collection, contains *Obſervations on the caſes publiſhed by* Mr. Blair, in a pamphlet, intitled, "*Eſſays on the Venereal Diſeaſe, and its concomitant Affections, illuſtrated by a Variety of Caſes. Eſſay I. Part I.* &c,"—In this ſection we find ſome ſevere ſtrictures, 1. on the "twenty-three experiments with the acid of nitre, the citric acid, and the oxygenated muriate of potaſh, in primary ſymptoms;" 2. "on twenty-ſix caſes of confirmed ſyphilis, wherein the acid of nitre was exhibited;" and, 3. on "eleven trials with the oxygenated muriate of potaſh, in advanced ſtages of the lues venerea." In all of theſe, the author's decided oppoſition to the new remedies, and his invincible predilection for *vetus amicus* mercury is ſo very conſpicuous, that his conduct, *on theſe occaſions*, requires no further comment or notice from us.

In the third and laſt diviſion of this work, Dr. Beddoes furniſhes us with his own "*Remarks on various Queſtions that have ariſen during the Inveſtigation of the antiſyphilitic Virtues of the Nitrous Acid.*" Theſe remarks are prefaced with extracts from the British Critic, and the Medical Review, relative to the above mentioned '*Eſſay*' of Mr. Blair's, and of which we ſhall only ſay, they are *in one ſtrain, and perfectly in character!*—As we have already declined entering into the merits of the controverſy, relative to the real or pretended effects of the nitric acid, we ſhall only add here, *en paſſant*, that in the teſtimonies adduced by Dr. Beddoes, there is ſuch a maſs of evidence in favour of this ſubſtance, as an excellent ſubſtitute for mercury, in almoſt every ſtage of lues (if the conſtitution of the patient, and other

‡ *Vide* pag. 130——132 of the ſame '*Collection*,' where three markable caſes of lues are related, in the two firſt of which it appears, that the nitrous acid performed the cure, '*without the uſe of mercury*.'

other collateral circumſtances be properly attended to) that Dr. Beddoes, as ſtanding on higher ground, may triumphantly and ſuccesſsfully direct the ſhafts of criticiſm, and pointed ſatire, againſt his feeble antagoniſt. He concludes this reſpectable 'Collection' with eleven additional documents, in an appendix, which, in our opinion, are ſufficient to remove every remaining doubt, with reſpect to the unfounded ſuſpicious, and illiberal aſperſions thrown on the ſubject of the preſent inquiry, by his invidious opponents.

*Caſes of the Diabetes Mellitus; with the Reſults of the Trials of certain Acids and other Subſtances in the Cure of the Lues Venerea:* By JOHN ROLLO, *M. D.* Surgeon-General, Royal Artillery. Second Edition, with large Additions. *London.* Dilly.

To diffuſe the knowledge of the new method of treating the diabetes, and to place this publication within the reach of a greater number of readers, the learned author has been induced to compreſs the preſent edition into one volume. In the preface, we meet with two intereſting letters from CHISHOLM, from the ſecond of which we ſhall extract the following paſſage: "The *nitrous acid* is a moſt ſafe and efficacious remedy in hepatic complaints of old ſtanding, in all *venereal obſtructions*, and diſeaſes depending upon them."

This valuable work is divided into two PARTS: the firſt contains "*Caſes of the Diabetes Mellitus, with a general view of the nature of the diſeaſe and its appropriate treatment; including a conciſe review of what has been written on the ſubject; an anſwer to ſome objections urged againſt the doctrine we have delivered; and chemical experiments on urine and ſugar.*" p. 17—477.

In this extenſive range of inveſtigation, we find much curious and intereſting matter, particularly in the very judicious and accurate chemical experiments, inſtituted to illuſtrate this ſubject; and Dr. Rollo concludes this part with the following reſult: "1. That ſugar conſiſts of carbone, hydrogene, and oxygene; and may be conſidered as a *pure* vegetable oxyde; 2d. That ſugar of milk is compoſed of the ſame principle, but contains more oxygene, and conſiderably leſs charcoal; 3d. That gum differs from ſugar, in containing, beſides carbone, hydrogene and oxygene, both lime and azote; 4th. That vegetable farina cannot be converted into ſaccharine matter, without the joint action of oxygene and water, the firſt of which appears to be abſorbed, and the laſt decompoſed during this proceſs; 5th. That when ſugar is deprived of its oxygene, or combined with other ſubſtances, it loſes its characteriſtic properties, and is no longer ſuſceptible of the vinous fermentation; 6th. That neither vegetable nor animal mucilages, in their pure ſtate, are ſuſceptible of this proceſs.

"From a review of the whole, the propriety of the different medicines which have been employed in diabetes, muſt be obvious, more particularly the pure alkalies, lime-water, and the different ſulphurates, all of which muſt counteract the formation of ſaccharine matter in the ſtomach.—We alſo readily ſee the neceſſity of a diet, conſiſting entirely of animal food, being the only one which does not furniſh oxygene, and that peculiar but ſimple combination of carbone and hydrogene, conſtituting the baſis of ſugar, and without which it cannot be produced."

The *ſecond Part* of this truly practical work preſents us with "*The Reſults on the trials of various acids, and ſome other ſubſtances in the treatment of the lues venerea:* by WILLIAM CRUICKSHANK, and other Surgeons of the Artillery." p. 481—625.—To this part the author has ſubjoined a *new* chapter, in which he gives a correct and circumſtantial narrative of of the trials made by Dr. WITTMAN ; a table containing a lift of the patients afflicted with the venereal difeafe, " who have been treated in the Royal Artillery Hofpital at Woolwich, by the new remedies ;" and fome additional remarks on the effects of the nitrous acid, the oxygenated muriate of pot-afh, &c. in the lues venerea, by Mr. CRUICKSHANK.— From thefe laft, we fhall felect the following pertinent and conclufive paffages :

" Eighteen months have now elapfed fince the firft cafes treated by thefe remedies have been cured ; and of the firft feventeen, which were more immediately under our own management, *not one has relapfed, nor have the fecondary fymptoms made their appearance in a fingle inftance.* That the difeafe has been completely eradicated, can therefore admit of no doubt.—In our firft trials, we confined ourfelves, in a great meafure, to primary affections ; but for fome time paft no diftinction has been made, and the fecondary as well as the primary fymptoms have been all treated on the fame plan.

" The total number which have now been cured in the Hofpital, fince the beginning of March, 1797, amounts to 155, as will appear from the table; of thefe 13 had the fecondary fymptoms of the difeafe. This fmall number of fecondary cafes proves, in a great meafure, the certainty and efficacy of this mode of treatment ; for, as Dr. Wittman employed thefe remedies in all venereal affections, whatever their nature might be, had the cures not been perfect, the fecondary difeafe muft have been very common ; befides, of the 13, three only could be afcribed to this cafe, and thefe were all afterwards cured by the oxygenated muriate of pot-afh. Of the remaining ten, 4 appeared to be the natural confequence and progrefs of the difeafe, and fix followed a courfe of mercury."— P. 619, 620.

" Of the different remedies employed, we formerly gave the preference to the oxygenated muriate of pot-afh, * and we are now more convinced of its fuperiority ; for there have been many cafes where it has fucceeded much better than the nitrous acid." P. 624.

" We do not prefume to account for the numerous *failures* which have been recorded, but fufpect that they are to be afcribed either *to fome irregularity or impropriety in the adminiftration of the remedies, or to a want of perfeverance and fteadinefs in the practitioner or patient.* It is alfo proper to remark, that in thefe failures, the remedies were too feldom varied, fo that when one did not anfwer *immediately*, it was dropped, and mercury had recourfe to. Now we are confident, that much of our *uniform fuccefs* has been owing to the method, which was very early adopted, of changing the preparation, whenever it feemed to produce no further effect on the difeafe or conftitution. In this way, a number of cures were quickly obtained by the oxygenated muriate of pot-afh, &c. where the nitrous acid, &c. had not fo immediately fucceeded."

*Effai*

* Mr. Cruickfhank judicioufly varied the proportion, as well as the combination of this fubftance with calomel, &c. in different patients. Thus he directed according to the circumftances of the cafe, and the greater or lefs degree of feverity manifefted by the difeafe in the conftitution of the patient, from 10 to 15 grains of the oxygenated muriate of pot-afh, four times a day ; *with* or *without* the addition of *calomel*, from one to two grains daily—befides which he directed the faturnine lotion to be applied in thofe cafes which required this external aid ; and his fuccefs has been fuch as might have been rationally expected from the fcientific and cautious prefcriber of thefe remedies.

*Eſſai ſur les Fièvres intermittentes, &c. An Eſſay on intermitting Fevers, the Action and Uſe of Febrifuges, particularly the Peruvian Bark.* By L. D. A. Bouffey, 8vo. 424 pp. (5 Liv.) *Paris.* Croullebois.

The principles here laid down are ſtated with preciſion, and throw new light on this branch of human maladies. The character of fevers is conſidered under their different appearance, and the relative value of febrifuges aſcertained. Bark itſelf, ſo juſtly conſidered as the beſt febrifuge we know, but which, is every day uſed in too empirical a manner, is here reduced to practical rules, ſuch as to make its exhibition more ſafe, and its ſucceſs more certain. The Author happily reconciles the chemical action of this medicine with its moſt ſenſible effects in the animal economy, and thence deduces practical rules for applying it with advantage.

*Memoria di* Luigi-Eustachio Polidori, *ſopro un tiſo contagiaſo, &c. Memoirs reſpecting the Cure of a contagious Typhous Fever lately prevalent in Tuſcany.* Piſa. 1798.

The method which the Author has ſucceſsfully practiſed in that diſorder is the ſubject of this memoir. The regimen he recommends, conſiſts in cordials, rich ſoups and broths, with toaſted bread, in ſmall quantities, but often repeated ; muſtard to be added three or four times a day; that ſubſtance being, according to Callitzen, preferable in this caſe to the bark. The ordinary beverage ſhould be generous wine, in ſmall quantities, to be progreſſively increaſed, adding three or four times in the day the cardiac confection of the Edinburgh Pharmacopæia ; at night a ſudorific bolus, with ten or twelve drops of laudanum in a glaſs of good wine; this doſe alſo to be gradually increaſed. Friction of the vertebræ, and likewiſe of the extremities, with the palm of the hand, applying the volatile liniment of the London Pharmacopæia ; a frequent renovation of pure air, and ſprinkling the ſick chamber with vinegar, are recommended ; and to abſtain as much as poſſible from the uſe of evacuating and debilitating remedies.— The Peruvian bark Dr. Polidori has uniformly found uſeleſs, and often hurtful ; but he has not explained the cauſes which could have produced ſo ſingular an effect. In another part of his diſſertation he informs us, that he has not employed bliſters; being apprehenſive of too much evacuation. With every reſpect for the abilities of the Author, we cannot conſider this aſſertion as accurate or ſcientific. The experience we have had of the operation of this remedy, does not permit us to entertain a doubt, that it acts as an immediate ſtimulus, and as ſuch. Dr. Polidori might have preſcribed it with ſafety, and probably with benefit to his patients.

*Oeuvres medico-chirurgales,* &c. " *Medical and Chirurgical Eſſays, containing Obſervations and Diſſertations on different ſubjects of Medicine and Surgery.*" By Collomb. 8vo. (6 liv. 10 ſols) *Paris.* Croullebois.

This is a work of great merit, and will form a valuable addition to a medical library. Among its important contents, are diſſertations on oſſification ; on the lymph, cancer, gout, the organs of ſight ; Miſs Stephens' medicine for diſſolving the ſtone in the bladder ; on perſons born deaf and dumb ; a number of details and obſervations on gravidity and delivery ; on gun-ſhot wounds in the lungs ; on cancer ; on the uterine polypus ; on the prolapſus uteri, and other objects deſerving the attention of the medical inquirer. It concludes with a ſhort treatiſe on venereal diſorders.

*Medical Inquiries and Observations:* Containing an Account of the Yellow Fever, as it appeared in Philadelphia in 1797; and Observations upon the Nature and Cure of the Gout and Hydrophobia. By Benjamin Rush, M. D. &c. Vol. V. *Philadelphia* 8vo. 236 pp. 1798. Dobson.

After having furnished an accurate account of the weather and diseases which occurred during part of the year 1795, and the whole of the year 1796; commented upon the supposed connection between pestilential epidemics and the occurrence of earthquakes, the eruptions of volcanos, the appearance of comets, meteors, &c.; and sketched a continued analysis of the weather and diseases, from the close of his former work on this subject, to the beginning of the epidemic which appeared in 1797, the learned author proceeds to inform us, that no premonitory symptom was observed, except the tooth-ach, which obviously and remarkably preceded the attack of the fever in several cases.

The Doctor next enters into a detail of the symptoms of the Yellow Fever, as they appeared in the blood-vessels, in the excretions, in the nervous system, in the senses, in the lymphatic system, in the skin, and in the blood. Many interesting and valuable observations occur here; but the mode of treatment, in general, does not appear to have been very different from that pursued in the epidemics of 1793 and 1794. Mercury administered to such extent as to produce ptyalism, was not a little depended upon; and the author lost only two patients in whom this effect was not produced.

*Observations on the Causes and Cure of Remitting Bilious Fevers. To which is annexed an Abstract of the Opinions and Practice of different Authors; and an Appendix exhibiting Facts and Reflections relative to the Synochus Icteroides, or Yellow Fever.* By William Currie, M.D. &c. *Philadelphia.* 8vo. 227 pp. 1798. Palmer.

After some cursory remarks on the "Causes of Bilious Fevers," and on the diffusion of "febrile miasmata through the air," Dr. Currie enters into a more minute discussion of the two following interesting questions:

"1. Is the Bilious Fever, or any other variety of the Remitting Fever, occasioned by marsh-miasmata, or the exhalations from putrid or putrifying vegetables, ever contagious?

"2. And was the Yellow Fever which occasioned such deplorable mortality in Philadelphia, in the summer of 1793, and which has appeared in other sea-port towns in the United States of America since that period, only a higher grade of the Bilious Fever, generated by the same causes?"—To both of these questions the Doctor gives a decided negative.

*A Treatise on the Autumnal Endemial Epidemic of Tropical Climates, vulgarly called the Yellow Fever,* &c. By J. B. Davidge, M. D. *Baltimore.* 8vo. 65 pp. 1798. Pechin.

Notwithstanding the inelegant and rather obscure manner of writing which pervades this treatise, the author evinces no inconsiderable share of sagacity, learning, and judicious reflection. When by habits of composition he shall have attained a proper degree of purity and perspicuity of language, we doubt not he will produce something at once creditable to himself, and useful to mankind.

*Treatise on the Yellow Fever; shewing its Origin, Cure, and Prevention.* By JOSEPH BROWNE. *New York.* 8vo. 31 pp. 1798. Argus-Office.

THIS author is one of those who undertake to analyse and indicate the precise state of the atmosphere necessary to produce the Yellow Fever, with other diseases of a similar nature and type. His general principle is, that this fever is produced by heat and a deficiency of oxygenous air, or, as he terms it, "*animal vital air*;" or a surcharge of azotic, or "*vegetable vital air.*" He considers the atmosphere as being principally a chemical compound of these two airs, and "when any portion of atmospheric air comes in contact with any substance that has a greater affinity with either of its component parts, than these parts have for each other, a decomposition of this portion takes place, and a new union is formed."

*Proceedings of the College of Physicians of Philadelphia, relative to the Prevention of the Introduction and spreading of contagious Diseases.* PHILADELPHIA. 8vo. 37 pp. 1798. Dobson.

THIS little pamphlet comprises all the proceedings of the College, in respect to the importation of diseases, from Aug. 1793 to Aug. 1797, in a very convenient form; and moreover exhibits the most essential parts of the various medical discussions which have taken place on this subject, since the first mentioned period, when the Yellow Fever broke out in Philadelphia.

*An Inaugural Dissertation on Cynanche Trachealis, commonly called Croup or Hives.* By JOHN ARCHER, Jun. *Philadelphia.* 8vo. 46 pp. 1798. Way and Groff.

THE discovery of a remedy for so violent a disease, certainly merits to be circulated in the medical world, by the earliest and most extensive channels of communication. The author contends that the seneka root does not operate a cure, merely from its emetic and diaphoretic properties, as he has in some instances seen it produce a complete cure, without having had any effect as an emetic, diaphoretic, or cathartic. "Does it not then (he asks) cure cynanche trachealis chiefly by acting as a *local stimulant?*"—"I would not," he adds, "give an ounce of seneka as a chance in the cure of croup, for all the *emetic tartar, mercury,* and *cantharides* in the United States."

As we have already, under the head '*Medical and Physical Intelligence,*' p. 83, given a particular account of Dr. Archer's method of treating this malignant disease, we refer the reader to that useful and practical information.

