# Erratum

**Published:** 1799-04

**Authors:** 


——102 *Dele* the second and third lines from the top of the page.

