# Errata

**Published:** 1799-05

**Authors:** 


—— 216, *in the laſt line, the reference to the* 102*d page of No. I. has accidentally been dropped in impreſſing the laſt ſheet; although it had been diſtinctly marked in the manuſcript, as well as in the proof-ſheet.*

